# What patients with advanced cancer experience as helpful in navigating their life with a long-term response: a qualitative study

**DOI:** 10.1007/s00520-024-08398-2

**Published:** 2024-03-12

**Authors:** Laura C. Zwanenburg, Marije L. van der Lee, José J. Koldenhof, Karijn P. M. Suijkerbuijk, Melanie P. J. Schellekens

**Affiliations:** 1 Scientific Research Department, Centre for Psycho-Oncology, Helen Dowling Institute, Professor Bronkhorstlaan 20, 3723MB, Bilthoven, The Netherlands; 2 Department of Medical and Clinical Psychology, Tilburg University, School of Social and Behavioral Sciences, Tilburg, The Netherlands; 3 Department of Medical Oncology, University Medical Centre in Utrecht, Utrecht University, Utrecht, The Netherlands

**Keywords:** Psychosocial functioning, Lung cancer, Melanoma, Immunotherapy, Targeted therapy, Qualitative research

## Abstract

**Purpose:**

Despite improved survival for people with advanced cancer due to new medical treatments, a growing group of long-term responders (LTRs) has to learn to live with uncertainties that affect several life domains. At the core of their experience, they neither feel like a patient nor feel healthy. Despite growing awareness of LTRs’ experiences, learning more about how they cope with their long-term response can provide insight into how to best support them. Our study aimed to gain a deeper understanding what LTRs experience as helpful in navigating life with a long-term response.

**Methods:**

We conducted an exploratory qualitative study using thematic data analysis. Semi-structured in-depth interviews were conducted with 17 participants with advanced melanoma or lung cancer with confirmed response or long-term stable disease while on immuno- or targeted therapy.

**Results:**

LTRs reported several strategies to navigate life with a long-term response, for example, by involving the social environment, seeing uncertainty as an opportunity, and being present in the moment. This helped them to *reclaim a sense of control*, *alter their perspective*, and *reshape their lives according to their values*.

**Conclusion:**

Using different coping strategies enables LTRs to acknowledge both their sick and healthy side. Striking a healthy balance between being oriented on feeling sick or feeling healthy can help LTRs and their close others to navigate life with a long-term response. Healthcare professionals can provide support by recognizing whether LTRs are oriented at feeling sick or healthy, and by actively involving close others during medical appointments.

Long-term responders (LTRs) are patients with advanced (i.e., metastatic) cancer who obtain durable survival due to effective treatment with new medical therapies such as immunotherapy (IT) or targeted therapy (TT) [1, 2]. These therapies seem promising for treatment of melanoma and lung cancer. For example, after effective treatment with IT, more than 20% of advanced lung cancer and up to 50% of melanoma patients in studies are still alive after respectively 5 [3] and 6.5 years [4]. While it is good news that treatment is effective in prolonging life, approximately half of these patients report heightened levels of distress due to their uncertain prognosis or fear of disease progression [5, 6]. LTRs feel they do not belong to people who die from cancer within the foreseeable future nor to people who receive(d) treatment with curative intent. LTRs often feel misunderstood by the social environment, experience the loss of a carefree life, and have to deal with ongoing uncertainty about life expectancy. Recurring control scans and self-monitoring their bodies can induce fear of disease progression. As such, LTRs struggle to adapt to a life with cancer [1].

Despite growing awareness and understanding of LTRs’ experiences [1, 7–13], there is a great need for further research on how to best support them navigating their life with a long-term response [14, 15]. A valuable starting point is to learn from LTRs themselves, exploring the strategies they employ and what they experience as helpful in dealing with the challenges that come with a long-term response. So far, studies have described the experiences of obtaining a long-term response, shortly describing the following strategies: LTRs try to regain a sense of control by monitoring their body [11], by ensuring that significant others will be taken care of in case the LTR dies, and by trying to keep life as normal as possible [9]. They try to maintain a positive outlook [10] and to focus on the present and what they find most important [8, 11].

In-depth insight into LTRs’ own strengths, abilities, and what they can do themselves is of utmost importance to prevent mental health problems and to support LTRs with patient-centered care. Qualitative research can provide this knowledge, which can be considered as a form of practical experience or know-how that patients already use in daily life. Therefore, our study aimed to gain a deeper understanding of what LTRs experience as helpful in navigating a long-term response.

## Materials and methods

### Study population

Participants were eligible when diagnosed with advanced lung cancer or melanoma and a confirmed response (i.e., at least two consecutive scans showing response to treatment) to or long-term stable disease while on IT or TT. Participants needed to be at least 18 years old and be able to speak and read Dutch.

### Recruitment

From April to June 2021, participants were recruited at Helen Dowling Institute (HDI), a mental healthcare institute for psycho-oncology, and the outpatient clinics of University Medical Centre in Utrecht and Radboud University Medical Centre in Nijmegen, the Netherlands. Eligible participants were identified by healthcare professionals (HCPs), provided with information about the study, and referred to the researcher. The researcher (LZ) contacted willing participants by phone to provide further study details and schedule an interview. To obtain a broad view on what LTRs found helpful, the researchers used purposive sampling (i.e., including patients with different cancer types (melanoma or lung cancer), treatments (IT or TT), genders, and age). All participants provided written informed consent before participating in the study. Recruitment of participants stopped when no new codes emerged from data.

### Semi-structured interviews

Semi-structured interviews were conducted by LZ, using an interview guide (Table 1) that was developed and pre-tested with input from a LTR. The interviews were conducted face-to-face at the HDI (*n* = 4), the participants’ residence (*n* = 3), or online via video connection (*n* = 11), and lasted between 41 and 69 minutes. The audio-recorded interviews were transcribed verbatim, and a brief summary of the interview was sent to the participant for a member check.

**Table 1:** Guide for semi-structured interviews

Topic	Questions
Opening	I would like to know how you are doing at the moment: *Can you tell me something about how you are feeling today?* *How are you doing physically?* *How are you doing mentally?*
Disease trajectory	I would like to know how the disease trajectory has been and how you experienced it: *Can you briefly tell me about your disease trajectory before you started IT/TT?* *Can you go back to when the oncologist suggested to start IT/TT?* *What were your thoughts about this therapy?* *Can you briefly tell me about your disease trajectory after you started IT/TT?* *What was it like when IT/TT turned out to be effective?*
Living longer than initially expected when getting an advanced cancer diagnosis	Due to a good response to IT/TT, patients live longer than initially expected. I would like to find out what you find helpful when adapting to a life with cancer and dealing with challenges brough along with living longer: *What was helpful in dealing with these challenges? Could you please elaborate?* *What would you advise other LTRs in dealing with these challenges? Could you please elaborate?* *What would you advise HCPs in helping LTRS to deal with these challenges? Could you please elaborate?* *How could your social environment help you? Could you please elaborate?*
Living with an uncertain life perspective	I would like to know how you are managing living with an uncertain life perspective: *What was helpful in managing uncertainty? Could you please elaborate?* *What would you advise other LTRs in managing uncertainty? Could you please elaborate?* *What would you advise HCPs in helping LTRS to manage uncertainty? Could you please elaborate?*
Closing	*Is there anything else you’d like to discuss that we haven’t already discussed?* *What was it like taking part in this interview?*

### Qualitative data analysis

Data were analyzed using MAXQDA software (Version 2022) and inductive thematic analysis according to the phases outlined by Braun and Clarke (Table 2) [16].

**Table 2:** Qualitative research phases according to Braun and Clarke [16]

Phase	Description	Researchers	Example
1. Familiarization	Listening to audio tapes, transcribing interviews, and reading transcripts	LZ, MS	-
2. Data coding	Coding interesting sentences, conducting and coding additional interviews until data saturation was reached	LZ, MS	“Reading information about treatment”
3. Generating initial themes	Discussing codes until reaching consensus, categorizing them into possible (sub)themes	All	“Trying to get a sense of control” and “Increasing knowledge about treatment”
4. Reviewing and developing themes	Discussing themes, checking themes with data	All	
5. Refining, defining, and naming themes	Discussing and refining themes	All	“Reclaiming a sense of control” and “Increasing knowledge about disease and treatment”
6. Writing the report	Writing the manuscript according to COREQ reporting guidelines	All	-

## Results

### Sample characteristics

Of the 21 invited patients, three were unwilling to participate due to logistic reasons, expected emotional burden, and an unknown reason. One participant was found to have no confirmed response to IT during the interview and was excluded from analysis. Seventeen participants were included for analysis (Table 3). Most participants were women and participants’ age ranged from 33 to 74 years old. Approximately 88% of participants was highly educated. The number of participants with melanoma and lung cancer was equally distributed. In line with expectations, three-quarters of the participants are/have been treated with IT and a quarter with TT because treatment with TT in melanoma patients in contrast to lung cancer patients is often only effective for a shorter period of time.

**Table 3:** Sociodemographic and clinical characteristics of the 17 patients

	*n*	Percent
Age, *M* (SD)	56.4	13.0
Gender		
Men	6	35
Women	11	65
Educational level		
Low	1	5.9
Intermediate	1	5.9
High	15	88.2
Working status		
Employed	8	47.1
Unemployed	1	5.9
Disabled	1	5.9
Sick	1	5.9
Retired	5	29.4
Volunteering	1	5.9
Relationship status		
Single	1	5.9
Married/living together	14	82.4
Divorced	2	11.8
Cancer type		
Lung cancer	9	52.9
Melanoma	8	47.1
Time since…, M (SD)		
Diagnosis *in months*	38.3	26.4
Start current treatment *in months*	27.7	18.5
Best response *in months*	22.4	15.9
Treatment		
Immunotherapy*	13	76
Ipilimumab	1	
Ipilimumab + nivolumab	1	
Nivolumab	4	
Pembrolizumab	6	
Targeted therapy	4	24
Afatinib	1	
Osimertinib	3	
Psychological guidance		
Before diagnosis	3	17.7
After diagnosis	9	52.9

*Data of one participant is missing

### Navigating life with a long-term response

Living long-term with advanced cancer and an uncertain life perspective required LTRs to adapt to a new situation which seemed to encompass two distinct roles: a sick role, in which cancer is very present (e.g., having medical check-ups or when others ask about cancer), and a healthy role, in which their illness is less prominent (e.g., when trying to resume life). Participants reported different strategies trying to navigate their long-term response and the uncertainty that follows from it. These strategies were clustered in three main themes: (1) *reclaiming a sense of control*; (2) *altering one’s perspective*; and (3) *reshaping daily life according to one’s values* (Table 4).

**Table 4:** Themes, subthemes, and quotes of navigating life with a long-term response

Themes	Subthemes	Quotes
1. Reclaiming a sense of control	1.1. Relying on the medical team and advances in medical science	*“I’m trying to keep in mind that the medical world is at the point where they can keep you alive for quite a couple of years. And that the focus should be on that, and not on being sick.”* – man, 72 years, lung cancer, IT
	1.2. Increasing knowledge about disease and treatment	*“But it really kept me occupied. I have read a lot about how the treatment works and what kind of treatment it exactly is. Yeah that’s my way of dealing with things like this. I’m working as a consultant, so it’s my job to quickly pick up on things I don’t understand yet and then understand it better than anyone else. For me, it’s also a way, emotionally, because of course it’s nonsense, or well, not complete nonsense, but it’s a way to gain emotional control over it.”* – man, 30 years, melanoma, IT
	1.3. Arranging practical matters around disease progression and dying	“*What really helped me in the beginning was thinking about what I have to arrange before I die? So, I made a list of addresses of people who could get a card and I started to divide my stuff, like that’s for him, that’s for her. I also told my children, a funeral can be very expensive, but be aware that it’s your party. It’s not for me. I’m there, but I’m not participating, so you can do it as you want. As far as I’m concerned, do it as cheap as possible, just put me in a basket or in a cloth. I think it’s all good and cremation will also do. They [children] can do it the way they want it.”*- woman, 74 years, lung cancer, TT
	1.4. Engaging in routine tasks	*“It also helped me a lot to keep dressing myself. I kept doing my daily things, the shopping, going to the playground. I get a lot of positivity from clothes and makeup and I just kept doing that. And sometimes I put on my sweatsuit with a bun on my head, my lenses off and glasses on, but I just do that when I’m home in the evening and when I know I won’t leave anymore. And otherwise I’m always on top, and that gives me a lot of positivity. And that’s why I chose to keep doing that.”* – woman, 33 years, melanoma, IT
	1.5. Involving the social environment	*“The disease has brought us closer together. It was my birthday last week and my kids came over. Then we talked about it. When I got sick a year ago, the kids really wanted to be with me and they were super sweet and caring and so was my husband. That was very beautiful.”* – woman, 55 years, lung cancer, TT
2. Altering one’s perspective	2.1. Not taking things for granted	*“It doesn’t always have to be so difficult or hard. I can also really appreciate the very small things and then really enjoy myself. Yes, I think that’s the beauty of this [getting an advanced cancer diagnosis] too. Maybe because it made me realize that life can suddenly end.”* – woman, 63 years, melanoma, IT
	2.2. Acknowledging dying is part of life	*“Which is also important that you realize that you’re not the only one who dies. It helped me too think, oh, I’m not the only one who dies, but everyone has to say goodbye to me and I have to say goodbye to everyone, but a thing I also think, maybe they will die earlier than me. Yes, a kind of equality, also equalizing your situation to others.”* – woman, 68 years, lung cancer, TT
	2.3. Sharing with and learning from fellow LTRs	*“What has been really helpful in recent years is contact with others. Fellows, online or in groups. Everyone deals with it differently, of course, but to be able to get something out of that contact could help me, that’s very nice. “Oh is that what you do? Is that how you approach it? Would that also be something for me?”*—man, 56 years, lung cancer, IT
	2.4. Seeing uncertainty as an opportunity	*“When everything is uncertain, anything is still possible. So, then it can also go well. And so yes, you have to be able to see the positive side. For example, I could just start working as an actress and then I could just win an Oscar.” –* woman, 47 years, melanoma, IT
3. Reshaping daily life according to one’s values	3.1. Evaluating life priorities	*“I stopped doing the work I had always done. I was like I want to slow down a bit. I’m really focusing on my health. I want to work, but I don’t want to do anything in the evening. In the evening I always did a lot for my work, I was never really done… And now I just want to work regularly and in the evenings I want to be at home or do other stuff.”* – woman, 61 years, melanoma, IT
	3.2. Being present in the moment	*“I just tried to enjoy the things that are here and that is being in nature, doing sports, being with family and friends, and experiencing that a bit more consciously than before perhaps. Before, you take it for granted… Life is now and not in a month’s time and we’ll see. Be happy with what you have instead of not being happy with what you could have.*” – man, 60 years, melanoma, IT
	3.3. Balancing rest and activity	*“You become very selective, because the fatigue plays tricks on you every time. You can only use your energy once of course. And then I prefer to put that into the things that are really important and the important people around me.” –* woman, 55 years, melanoma, IT
	3.4. Setting short- and long-term goals	*“But yes the future, of course I want to have a walker race with my girlfriends, yes I want to do that. I want to meet my child’s husband or wife, I want to see her get married. Yes, I do have goals that I definitely want to achieve. And I think that’s good too, that you set goals for yourself.”* – woman, 33 years, melanoma, IT
	3.5. Adapting activities in response to limitations	*“I can’t do a lot of things anymore. For example, working in the garden. My great passion, I can’t work in the garden anymore. I need help with it. I can sow, I can fertilize and I do that. Then they put that sack of manure in front of me with a shovel, like I’m half an invalid, but I think I’ll do it anyway.”* – woman, 70 years, lung cancer, IT

### Theme 1: Reclaiming a sense of control

Participants described that upon hearing the diagnosis, cancer completely took over their lives. While facing an uncertain future, LTRs experienced a loss of control, which posed a tremendous challenge. LTRs attempted to preserve their autonomy by reestablishing a sense of control, using various strategies.

#### Relying on the medical team and advances in science

Concerning this uncertainty and loss of control, LTRs identified good and personalized medical care as highly important. That is, HCPs communicated openly and honestly, had personal attention and tried to see the whole person instead of just a cancer patient, provided space for shared decision-making, and made time to recognize and normalize LTRs’ emotions and concerns. After starting treatment, trusting their HCPs helped LTRs to give in to their sick role. As time passed, LTRs felt more confident to take on their healthy role because they felt assured of receiving adequate care in case of disease progression. Furthermore, advances in medical science appeared to give many LTRs confidence that new treatments would be available should resistance occur.

#### Increasing knowledge about disease and treatment

LTRs often struggled to comprehend why they had developed cancer, for example because they had rarely been ill in the past and believed they had a healthy lifestyle. To gain a sense of control, LTRs strived to increase their understanding of the disease and treatment, ensuring that they correctly comprehended all medical information.

#### Arranging practical matters around disease progression and dying

Prompted by their sick role to create some certainty amidst their uncertain prognosis, LTRs arranged practical matters related to potential disease progression and dying. This involved exploring alternative treatments or clinical trials, creating a will, ensuring that financial matters (e.g., a mortgage) were arranged, arranging a declaration for euthanasia, and considering funeral arrangements. Arranging these matters themselves increased LTRs’ sense of autonomy. Together with knowing that things were settled, this could provide a sense of control, which allowed LTRs to let go of their sick role.

#### Engaging in routine tasks

Frequently, LTRs found it helpful trying to keep life as normal as possible. Some participants described engaging in familiar tasks (e.g., housekeeping) and staying occupied could serve as a source of comfort during times of uncertainty. Other participants described getting dressed properly instead of keeping their pajamas on helped them shift from the sick to the healthy role.

#### Involving the social environment

Due to the complexity of the situation, the social environment often found it difficult to understand what LTRs had to endure. In response to this, LTRs actively considered whether they wanted to share their feelings or updates about the disease with others. Some LTRs chose not to share this to shield their social circle from their difficult situation, as well as to protect themselves from others’ reactions. As a result, LTRs sometimes felt lonely. When they did choose to share their concerns with others, they often felt supported, which fostered deeper connections.

### Theme 2: Altering one’s perspective

LTRs described that when having negative thoughts about their disease and uncertain future, it could be beneficial to adopt a broader perspective. In particular when LTRs experienced feelings of despair, the social environment could be of major help to put things in perspective.

#### Not taking things for granted

Previously, some LTRs took being healthy for granted. Being diagnosed with advanced cancer caused not only sadness, but also a sense of unfairness, making LTRs question why this had happened to them. Discovering that treatment was available and effective shifted their perspective. LTRs experienced a sense of gratitude and viewed themselves as lucky, thereby facilitating their transition away from the sick role. Living longer with cancer made them realize that being healthy should not be taken for granted.

#### Acknowledging dying is part of life

Being diagnosed with a life-threatening disease confronted LTRs with the finiteness of life. LTRs mentioned that it was difficult for them when it appeared that others did not acknowledge the seriousness of their situation, for example, by saying “but I could also get run over by a truck tomorrow.” On the other hand, LTRs would sometimes say to others “but you could die too” making themselves equal to others and shifting from the sick to the healthy role. LTRs explained how discussing death with their social circle could help alleviate the heaviness of this topic and acknowledge that dying is a part of life.

#### Sharing with and learning from fellow LTRs

Although some LTRs did not want to be burdened by negative experiences of others, other LTRs found that sharing their experiences with peers was very helpful. When connecting with peers, a sense of mutual understanding was fostered and made them feel less alone. Through discussing their experiences and sharing advice, LTRs learnt from each other and gained a broader perspective on their situation.

#### Seeing uncertainty as an opportunity

Many LTRs found it challenging to live with uncertainty, as disease progression could potentially be detected at every check-up. However, few LTRs indicated that when everything is uncertain, everything is possible. Hence, uncertainty could also be seen as a kind of openness, creating space for optimism. While being overly optimistic led to denial of the gravity of their situation, it also served a functional purpose. Believing that things would turn out okay provided LTRs with the motivation to endure challenging medical treatments and check-ups.

### Theme 3: Reshaping life according to one’s values

Due to their uncertain life perspective, many LTRs struggled to adapt to a life with cancer. While looking for some direction in their lives, LTRs employed various strategies which could help them to reshape their life according to their values.

#### Evaluating life priorities

Their altered physical condition forced LTRs to evaluate their priorities. LTRs started to organize their lives in alignment with their values. For example, while initially building a career might have been crucial, LTRs prioritized spending time with close others after their diagnosis. By consciously dedicating their time to people and activities that hold significance, LTRs directed their own lives.

#### Being present in the moment

LTRs considered living in the moment as a helpful coping strategy for dealing with the uncertainty of the future. Taking things day by day prevented LTRs from becoming overwhelmed by the fears associated with their disease. Furthermore, being present in the moment allowed patients to cherish joyful moments during challenging times.

#### Balancing rest and activity

Due to their altered physical condition, LTRs needed to find a new balance in life. Participants reported that when they felt energized, they felt more like they were healthy and worried less about disease progression. The absence of fatigue was commonly mentioned as a key component of the healthy role and an important contributor to feeling well. Participants made a conscious effort to regulate their energy levels by maintaining a strict schedule between activity and rest as feeling fatigued could hinder engaging in meaningful activities.

#### Setting short-term and long-term goals

LTRs indicated that it helped them to keep setting goals that match their values. In the sick role, it could help to set short-term goals, such as planning to go on a holiday this summer, because this could give direction to life. As part of their healthy role, LTRs could envision a future and established long-term goals, such as wanting to attend major life events in their child’s life (e.g., graduation). The will to achieve goals helped LTRs to keep living their lives.

#### Adapting activities in response to limitations

LTRs emphasized the importance of persisting in activities despite limitations imposed by the disease. After feeling down about the inability to engage in certain activities, LTRs realized that by modifying their activities to match their capabilities, they could still derive joy from these pursuits (e.g., assisting in the classroom instead of being the main teacher). LTRs’ awareness of their limitations initially confined them to the patient role, but adapting their activities helped them to transition to the healthy role.

## Discussion

Our study provides insight into what LTRs find helpful in navigating life with a long-term response. Each LTR reported a number of different strategies. By alternating between different strategies, LTRs gradually learned what strategies were helpful to apply in different situations. LTRs indicated that these strategies were often not deliberately applied and usually occurred automatically. Using different strategies, such as *engaging in routine tasks*, *sharing with and learning from peers*, and *evaluating life priorities*, provided space for both feeling sick and healthy and helped LTRs to alternate between their sick and healthy roles. This enabled them to *reclaim their sense of control*, *alter their perspective*, and *adapt their lives according to their values*, and ultimately to navigate their life with a long-term response.

The duality of living with an uncertain cancer prognosis has previously been found in several studies among advanced cancer patients. These studies described, for example, a double awareness, where patients are simultaneously engaging with life while facing the reality of death [17]. The duality has also been described as a contrast between patients’ desire to live normally while also being aware of the possibility of death [18, 19] a dialectical movement between existential suffering and existential health [20] and as living in the tension between life and death [21]. These findings may suggest that LTRs experience a continuous demand to hold a degree of flexibility in employing diverse coping strategies that match their different roles. According to Masten [22] and colleagues, the effectiveness of coping strategies is not solely reliant on one’s skills. Rather, it involves a multifaceted interplay of behavioral, cognitive, emotional, physical, and social factors influenced by the context (e.g., phase of disease and sick or healthy role) as well as type and intensity of the stressors. LTRs need to engage in various strategies when embodying or shifting between the sick and healthy roles. This can be complicated by the roles being new and unfamiliar and the duality of these roles.

A framework that may provide further insight into the duality LTRs experience is the Dual Process Model of Coping with Bereavement (DPM). This seems relevant as many LTRs are grieving their former self and the life they envisioned [1, 7, 11, 13, 23]. The DPM describes the grieving process as a pendulum swing, alternating between being loss-oriented (i.e., coping with the loss experience itself and processing it) and restoration-oriented (i.e., coping with the changes brought about by the loss) [24]. A fundamental aspect of adaptive coping entails oscillation, which is the requirement to shift back and forth between both orientations [24]. In a similar vein, it may be beneficial for LTRs to oscillate between the sick role (i.e., loss-oriented) and healthy role (i.e., restoration-oriented). Our findings highlight that LTRs employed different strategies that match their orientation. For example, when LTRs are oriented on feeling sick (e.g., at times of control scan), they found it helpful to apply strategies such as *arranging practical matters around disease progression and dying*. When LTRs were more oriented on feeling healthy, strategies such as *balancing activity and rest* were considered helpful. In addition, LTRs applied other strategies that facilitated the process of oscillation between these orientations, such as *seeing uncertainty as opportunity.*

Of great importance for the process of oscillation is the involvement of close others, as they need to shift with the LTR between both orientations and are involved in strategies. For example, LTRs and their close others are alternating between sharing pain and grief (sickness-oriented) and continuing everyday family life as much as possible (health-oriented) [25]. Protective buffering or shielding the other from distress by concealing one’s own worries can complicate the joint process of oscillation and has adverse effects on the relationship between LTRs and their close others [26, 27]. When LTRs choose not to share their concerns, it can be difficult for the close others to align their support with the specific needs associated with LTRs’ orientation in that moment. This emphasizes the importance of open communication when navigating a long-term response.

### Strengths and limitations

The majority of the interviews took place online. While this method can pose challenges in terms of sharing vulnerabilities [28], LTRs seemed to readily open up and discuss their experiences with the interviewer, displaying various emotions throughout the interviews.

Nearly 90% of participants were highly educated, limiting the generalizability of our findings. Lower educated patients use cognitive avoidance more often as a coping strategy and engage less in activities such as reading medical information or studying statistics compared to higher educated patients [29, 30]. The findings indicate considerable variability in both active and passive strategies. Consequently, it is plausible that some of these strategies may be relevant for lower-educated LTRs as well.

The focus of the study on participants with melanoma or lung cancer limits the findings’ applicability to all LTRs. However, our findings reveal no strategies that are specifically linked to the clinical picture of melanoma or lung cancer. Living with uncertainty emerges as a shared element that potentially influences experiences to a greater extent than cancer type.

### Implications for research and clinical practice

When LTRs are in need of professional help and apply for psychological support, a promising treatment which can inspire therapists and LTRs is Acceptance and Commitment Therapy (ACT). The present findings showed that LTRs need flexibility to engage in different coping strategies. ACT specifically aims at cultivating psychological flexibility, which is described as being fully in contact with the present moment and being capable of flexibly engaging in behavior which aligns with personal values [31]. ACT includes exercises that can help LTRs to *alter their perspective* and *reshape daily life according to their values* [32].

It is important for HCPs to be aware and to recognize whether their patients are oriented on feeling sick or healthy, so they can tailor their care. For example, when LTRs are sickness-oriented, HCPs can make use of Advanced Care Planning to encourage their patients to *arrange practical matters around disease progression and dying*. Consequently, this can empower LTRs to *reclaim a sense of control* [33].

Close others can be of great support for LTRs; however, our study showed that communication can be very difficult due to a lack of understanding or protective buffering. By actively involving close others, HCPs can contribute to a better understanding, for instance by communicating the importance of coming along to medical appointments and asking whether close others understand all provided information. This could help prevent protective buffering, and enhance the experience of social support among LTRs.

Although it is known that close others also experience difficulties with adapting to a life where uncertainty is constantly present [9], research into the experiences and needs of LTRs’ close others is sparse. Studying and understanding the needs of LTRs’ close others is a crucial element in tailored care and support for LTRs as well as their close others.

## Conclusion

Effective treatment with IT and TT not only means an increased survival for advanced cancer patients, but also comes with many challenges such as facing an uncertain life perspective and fear of disease progression. In response to this, LTRs are actively finding ways to *reclaim a sense of control*, *alter their perspective*, and *reshape their life according to their values.* LTRs may benefit from applying different coping strategies that address both sides in themselves (i.e., sick role and healthy role), while including their close others in this process for optimal social support. Striking a healthy balance between being oriented on feeling sick or feeling healthy can help LTRs and their social environment to navigate life with a long-term response.

## Data Availability

The datasets generated and/or analyzed in the current study are not available publicly as eligible patients were informed before start of the interviews that their data would be stored securely and confidentially.
